# Insufficiency of B vitamins with its possible clinical implications

**DOI:** 10.3164/jcbn.20-56

**Published:** 2020-06-09

**Authors:** Kiyoshi Tanaka, Misora Ao, Akiko Kuwabara

**Affiliations:** 1 Faculty of Nutrition, Kobe Gakuin University, 518 Ikawadanicho-Arise, Nishi, Kobe 651-2180, Japan; 2 Faculty of Health and Nutrition, Osaka Shoin Women’s University, 4-2-26 Hishiyanishi, Higashiosaka, Osaka 577-8550, Japan; 3 Department of Clinical Nutrition, Graduate School of Comprehensive Rehabilitation, Osaka Prefecture University, 3-7-30 Habikino, Habikino, Osaka 583-8555, Japan

**Keywords:** vitamin insufficiency, osteoporotic fracture, cardiovascular disease, heart failure, hyperhomocysteinemia

## Abstract

Vitamin deficiency causes classical deficiency diseases such as beriberi and rickets. Vitamin insufficiency, which is milder than deficiency, is a risk for various chronic diseases, but its significance has not been recognized in Japan. Vitamin D insufficiency is quite common in Japan, and a serious risk for osteoporotic fracture through its unbeneficial effect on bone and muscle. Insufficiency of B vitamins has been little studied. However, hyperhomocysteinemia caused by the insufficiency of vitamin B_12_ or folate is a risk for cardiovascular disease, osteoporotic fracture, and cognitive impairment. Additionally, we have recently reported that vitamin B_1_ insufficiency is a risk for heart failure in the elderly. The effect of improvement of nutritional status including vitamins is less marked compared to drug treatment, but it costs far less, and is suited for the primary prevention of diseases. Randomized controlled trial is considered the study with the most robust evidence in the evaluation of drug treatment, but more emphasis should be put on the well-designed cohort studies in evaluating the role of nutrients. Vitamin insufficiency is quite prevalent, and vitamin requirement is much higher for its prevention than for the prevention of deficiency.

## Introduction: Vitamin Deficiency and Insufficiency

Non-communicable diseases (NCDs) or lifestyle related diseases are serious health concerns in Japan. Much emphasis has been put on the risk of the diagnosis, prevention, and treatment of NCDs. For their prevention, life-style modification can play important roles. Then, disease risk reduction is of utmost importance, and the significance of vitamins in the health promotion must be considered from such viewpoint.

Vitamin deficiency causes classical deficiency diseases. Examples with the responsible vitamins in the parentheses include beriberi (vitamin B_1_), pellagra (niacin), scurvy (vitamin C), rickets and osteomalacia (vitamin D), and coagulation abnormality (vitamin K).^(1)^ These severe deficiency diseases are generally considered to be mostly overcome in Japan, and the significance of vitamins in health promotion does not seem to be receiving much attention any more. Recently, however, vitamin insufficiency has been receiving increasing concern. Vitamin insufficiency, which is milder than deficiency, does not cause the classical deficiency diseases described above, but is associated with the increased risk of various diseases. Patients with vitamin deficiency have typical phenotypic changes, and can be individually diagnosed. In contrast, although vitamin insufficiency is related to the increased disease risk, it is not accompanied by the phenotypic abnormalities in each subject (Table 1). Therefore, it cannot be diagnosed individually, and tends to receive little attention.

Much more amount of vitamin is needed for the prevention of insufficiency than that of deficiency. “How much vitamins do we need for health promotion?” is a fundamental question, and the answer is greatly influenced by the indices based on which we define the required vitamin amount; deficiency or insufficiency. Considering the social background that NCDs are the leading cause of morbidity and mortality in Japan, we have considered it necessary to define the vitamin requirement based on the disease risk.

Of the various vitamins, vitamin D has received the highest concern regarding its insufficiency, and some description on vitamin D deficiency is given before going into the insufficiency of B vitamins. Of the B vitamins, those related to hyperhomocysteinemia (HHcy) such as vitamin B_12_ or folate will be described first, since there have been some publications on their relationship with disease risk. Additionally, we will make some discussion on the possible relationship between vitamin B_1_ insufficiency and disease risk, which, however, has been little studied.

## Vitamin D

The most fundamental action of vitamin D is to enhance the intestinal absorption of calcium and phosphorus. Since bone is formed in such a way as the calcium phosphorus deposition (mineralization) onto the matrix formed mainly by collagenous protein, vitamin D deficiency causes the mineralization defect; rickets in infants and osteomalacia in adults.^(2)^ Bone has two important roles; one as a supporting organ of the body, and the other as the calcium reservoir for maintaining the blood calcium concentration. Thus, in vitamin D insufficiency, intestinal calcium absorption is impaired. In order to maintain the blood calcium level, secondary hyperparathyroidism occurs, which in turn, increases bone resorption, leading to the increased risk of osteoporotic fracture.^(3)^

**Fig. 1:**
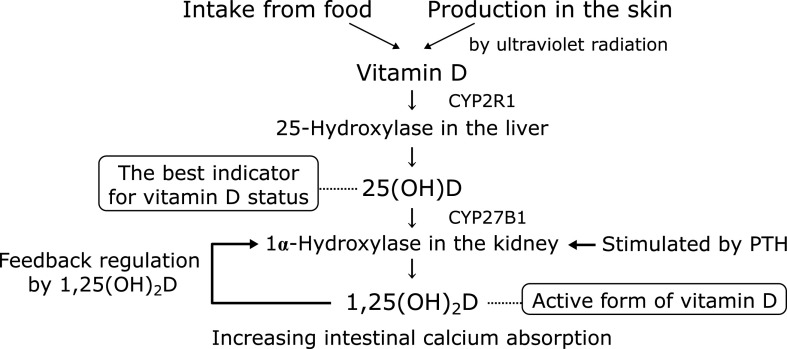
Metabolism of vitamin D.

Metabolism plays an important role in vitamin D action. Vitamin D, either from food or dermal production, undergoes two hydroxylation; first metabolized to 25-hydroxy vitamin D [25(OH)D] in the liver by such enzymes as CYP2R1, then catalyzed by CYP27B1 to 1,25-dihydroxy vitamin D [1,25(OH)_2_D] in the kidney, which is the active form of vitamin D.^(4)^ CYP27B1 activity is under the strict control; i.g. induction by parathyroid hormone (PTH) and suppression by hypercalcemia or increased serum level of 1,25(OH)_2_D (Fig. 1).

Serum 25(OH)D level is the best indicator of vitamin D status. Recently a guideline was published regarding its judgement as below.^(5)^

Sufficiency: Serum 25(OH) level, equal to or higher than 30 ng/ml

Insufficiency: Serum 25(OH) level, between 20 and 30 ng/ml

Deficiency: Serum 25(OH)D level, less than 20 ng/ml

Recently, other reference values for serum 25(OH)D levels have been described as below: sufficiency being defined as 25(OH)D >20 ng/ml (>50 nmol/L), insufficiency when the 25(OH)D are between 12 and 20 ng/ml (30–50 nmol/L), and deficiency when the 25(OH)D is <12 ng/ml (30 nmol/L).^(6)^

## Vitamin D Insufficiency and the Risks for Fracture and Falling

In a meta-analysis of 11 observational studies (39,141 participants, 6,278 fractures, 2,367 hip fractures), each increase of 10 ng/ml (i.e., 25 nmol/L) in 25(OH)D concentration was associated with an adjusted relative risk (RR) for any fracture of 0.93 (95% CI, 0.89–0.96) and an adjusted RR for hip fracture of 0.80 (95% CI, 0.75–0.86). Daily supplementation with both vitamin D and calcium reduced a 6% risk of any fracture (RR, 0.94; 95% CI, 0.89–0.99) and a 16% risk of hip fracture (RR, 0.84; 95% CI, 0.72–0.97).^(7)^ In contrast, no intervention studies with vitamin D have been conducted, and the numbers of cohort studies are limited in Japan. In Nagano cohort involving 1,470 community-dwelling post-menopausal women (63.7 ± 10.7 years old) for the average of 7.2 years, the risk of long bone and hip fracture was lower with the increasing serum 25(OH)D level, and the cut-off value of 25 ng/ml was obtained.^(8)^ Compared to the group with higher serum 25(OH)D level, the RR of fracture in the lower group was 2.71 (95% CI: 0.94–7.83, *p* = 0.07) for hip fracture, and 2.20 (95% CI: 1.37–3.53, *p*<0.01) for the long bone fracture. Thus, vitamin D deficiency was identified as the risk for non-vertebral fracture.

Recently, vitamin D is known to have many important actions in organs other than bone. Of these, its association with muscle strength is receiving much concern. In USA or Europa, many observational and intervention studies are available regarding vitamin D insufficiency as the risk of fracture and falling.^(9,10)^ One meta-analysis has reported that vitamin D supplementation has a small positive impact on muscle strength.^(11)^ Another one has shown that there was no improvement in muscle strength after the administration of vitamin D with or without calcium supplements in community-dwelling older persons.^(12)^ Thus, the effect of vitamin D on muscle has been controversial. We have recently reported that compared with the vitamin-sufficient counterpart, vitamin D deficient/insufficient subjects had lower skeletal muscle index (SMI) and muscle strength, and serum 25(OH)D level is a significant contributor to SMI and lower muscle strength.^(13)^

Of the osteoporotic fractures, most non-vertebral fractures occur at falling. Therefore, fracture prevention could be achieved both through increasing bone strength and preventing falls. In a meta-analysis, serum 25(OH)D concentration exhibited U-shaped association with decreased risk of falls and medium doses (50 to 100 µg/day) of daily vitamin D decreased falls in elderly women.^(14)^ Then, vitamin D deficiency/insufficiency is likely to increase the fracture risk via two mechanisms; impaired bone strength and increased risk of falling. Unfortunately, however, vitamin D intervention studies with the risk of falling and fracture as the endpoint have not been done in Japan.

In a cohort study with falling as the endpoint including 1,393 subjects 75 years old or higher, the odds ratio of falling was higher in subjects with their serum 25(OH)D level <20 ng/ml compared with those with their serum 25(OH)D level equal to or higher than 25 ng/ml.^(15)^ Thus, vitamin D deficiency/insufficiency is likely to increase the fracture risk both through the unfavorable skeletal effects and increased risk of falling.

Additionally, in the observation studies, that vitamin D insufficiency has been reported to be associated with the increased risk of other diseases including coronary heart diseases, cancer, and upper respiratory tract infection, and even with mortality.^(16)^

## The Requirement of Vitamin D Intake (Dietary Reference Intakes 2020 in Japan)

Recently, high prevalence of vitamin D deficiency/insufficiency has been reported in many articles worldwide including in Japan. In Research on Osteoarthritis/osteoporosis Against Disability (ROAD) study including 1,683 subjects (595 men and 1,088 women), percentage of subjects with their serum 25(OH)D level less than 30 ng/ml was as high as 81.3%,^(17)^ and it was even higher in women. It has repeatedly been reported that prevalence of vitamin D insufficiency is high in both genders and in all age groups, especially in women.^(17–19)^ High percentage of subjects with vitamin D deficiency/insufficiency is a world-wide problem, and even called an epidemic.^(20)^

**Fig. 2:**
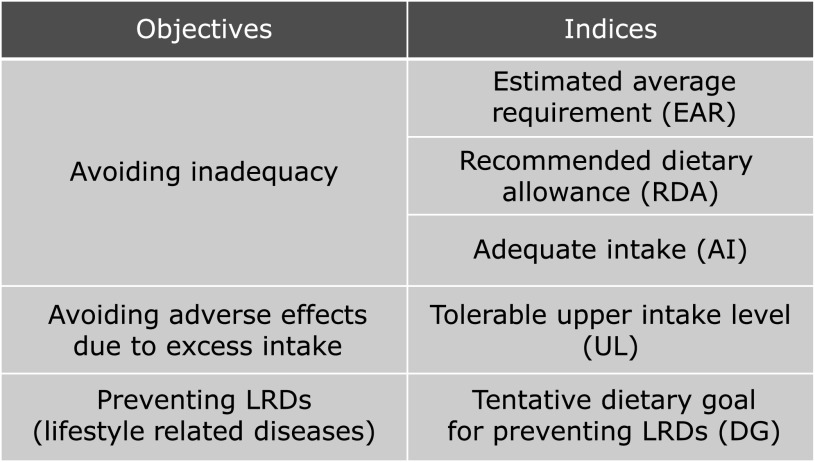
Indices employed in DRIs (dietary reference intakes) for Japanese (classified).

Requirements of energy and nutrients are described in Dietary Reference Intakes for Japanese (DRIs), which is revised every five years. In DRIs, five indices are determined for nutrients with three purposes; avoidance of insufficiency, avoidance of excess intake, and primary prevention of the life-style related diseases (Fig. 2).^(21)^

Estimated Average Requirement (EAR), Recommended Dietary Allowance (RDA) and Adequate Intake (AI) are the indices for the avoidance of nutrients insufficiency. EAR and RDA refer to the nutrient intake corresponding to the 50% and 2.5% probability of insufficiency. When robust evidence is unavailable for the determination of EAR and RDA, AI is decided instead. Tolerable Upper Intake Level (UL) is for the avoidance of unfavorable consequence of excessive nutrient intake. Tentative Dietary Goal for Preventing Life-style Related Disease (DG) is aimed for the primary prevention of life-style related diseases. In the current DRIs, diabetes, hypertension, dyslipidemia, and chronic kidney disease are the target of DG.

AI has been determined for vitamin D. In DRIs 2015, AI for vitamin D was 5.5 µg/day for adults, the basis of which was the most popular way to define the AI; the median intake of healthy subjects.^(22)^ In contrast, 10 to 20 µg/day intake is recommended in the Japanese guideline for the prevention and treatment for osteoporosis,^(23)^ and RDA in the DRIs in USA/Canada 2001 is 15 µg/day for subjects equal to or under 70 years old, and 20 µg/day for those aged 71 and over.^(24)^ Such apparent discrepancy is due to the different basis for the determination. In DRIs 2015, AI for vitamin D is based on the median intake of healthy subjects,^(22)^ whereas the basis is the prevention of osteoporotic fractures in the Japanese guideline for the prevention and treatment for osteoporosis,^(23)^ and DRIs in USA/Canada 2001.^(24)^ Recent studies have clarified that most Japanese are vitamin D deficient/insufficient. AI can be determined as the median intake when most subjects in the population have enough intake. Then, AI for vitamin D could not be defined as the median intake anymore for DRIs 2020. In the recently published DRIs 2020, AI was defined as the amount necessary for fracture prevention (15 µg/day) subtracted by the amount produced in the skin.^(21)^ The latter is greatly dependent on the season and latitude. In the DRIs 2020, 5 µg/day was adopted as the amount which can be produced even in Sapporo with high latitude during the winter time.^(25)^

Another problem in the determination of DRIs has been the lack of data with the simultaneous evaluation of vitamin D intake and serum 25(OH) level. In our previous study involving the institutionalized subjects with limited chance of going outside, vitamin D intake was 7.0 ± 1.4 µg/day which is much higher than the AI of 5 µg/day at that time. However, serum 25(OH)D concentration was as low as 11.1 ± 3.1 ng/ml and less than 20 ng/ml in practically all subjects; i.e. they were vitamin D deficient despite vitamin D intake far exceeding the AI.^(26)^ Intervention with 5 µg/day of vitamin D little improved the vitamin D status,^(27)^ and only in 40% of the subjects, serum 25(OH)D level exceeded 20 ng/ml after intervention with 20 µg/day of vitamin D.^(28)^ The implication of this data would be twofold. First, more vitamin D would be needed in the institutionalized elderly than the AI. The other is the importance of simultaneous determination of intake and the measurement of blood levels, which has not been done in the previous studies in Japan.

## B Vitamins Associated with Hyperhomocysteinemia

Vitamin B_12_ is involved in two enzymatic reactions as a co-enzyme; methionine synthase and methyl malonyl-CoA mutase. Pernicious anemia and subacute combined degeneration of spinal cord (SCDC) are the examples of its deficiency.^(29,30)^ Megaloblastic anemia occurs due to the deficiency of vitamin B_12_ or folate, and the deficiency of vitamin B_12_ leads to the damage to the nervous system. The classical clinical symptoms of B_6_ deficiency are a seborrheic dermatitis, microcytic anemia, epileptiform convulsions, and depression and confusion.^(31)^

Compared to the insufficiency of fat-soluble vitamins such as vitamin D, far less attention has been paid on the insufficiency of B vitamins. However, the significance of vitamin insufficiency is not limited to fat-soluble vitamins and the association between the insufficiency of B vitamin and disease risk is also an important issue.^(32)^ First, we would like to discuss the significance of HHcy, since it has received much attention within the context of B vitamins insufficiency.

**Fig. 3:**
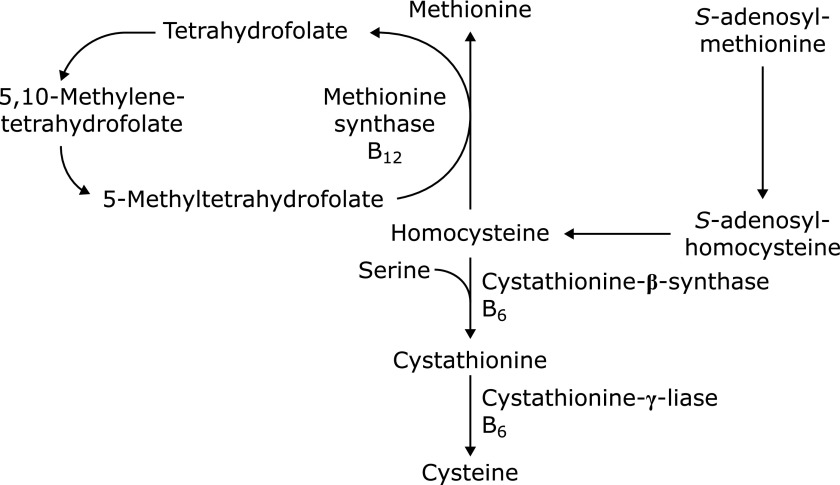
Methionine metabolism and Hcy.

Folate, vitamin B_12_, vitamin B_6_, and vitamin B_2_ are involved in one-carbon metabolism in their co-enzyme forms (Fig. 3).^(32)^ Transferring one-carbon unit from serine to tetrahydrofolate vitamin B_6_-dependently yields 5,10-methylenetetrahydrofolate, which is then converted to 5-methyltetrahydrofolate in a vitamin B_2_ (flavin adenine dinucleotide) dependent way. Homocysteine (Hcy) is an intermediate in the methionine cycle, and metabolized either by re-methylation to methionine or to cysteine by trans-sulfation. Re-methylation to methionine is catalyzed by methionine synthase with 5-methyltetrahydrofolate as a methyl group donor and vitamin B_12_ (methylcobalamin) as a cofactor. Alternative fate of Hcy is the trans-sulfation to cysteine in a vitamin B_6_-dependent way.^(32)^ Therefore, inadequate status of above-mentioned vitamins causes elevated plasma Hcy concentration; HHcy.

HHcy has been reported to exert various detrimental effects on the endothelial cells such as interference in nitric oxide production, deregulation of the hydrogen sulfide signaling pathway, oxidative stress, disturbance in lipoprotein metabolism, protein homocysteinylation, and cellular hypomethylation.^(33)^ As such, HHcy has been reported to be associated with the risk for CVD.^(34–38)^ Recently, updated systematic review from Cochrane Library has been published on the effects of Hcy lowering intervention with B vitamins for the prevention of CVD.^(39)^ A meta-analysis including 44,224 participants from 10 RCTs has shown a reduced risk for stroke with relative risk of 0.90 (95% CI 0.82–0.99). However, there was no significant effects regarding the risk of acute myocardial infarction or death from any cause.^(39)^

Recently a systematic review has been published on the effects of folic acid intervention with stroke as the outcome.^(40)^ This paper is characterized by that more than half of the participants were from the countries without mandatory fortification. Additionally, another meta-analysis on the folic acid intervention on the risk of stroke was published taking the factors possibly affecting the results into account.^(41)^ As a total, folic acid supplementation significantly reduced the stroke risk (RR 0.89, 95% CI 0.84–0.96), and the effect was greater in low folate status regions (Asia, RR 0.78, 0.67–0.90) compared to high folate status regions (America, RR 1.05, 0.90–1.23). Likewise, RR was 0.85 (95% CI 0.77–0.94) in regions without folic acid fortification, whereas it was 1.05 (95% CI 0.90–1.23) in fortified regions. Also, larger beneficial effects were observed with lower baseline vitamin B_12_ levels. The authors have concluded that folic acid supplementation could reduce the stroke risk in regions without folic acid fortification, particularly with low vitamin B_12_ levels. Taken these reports together, HHcy is a risk for CVD, especially stroke, and intervention by vitamins could effectively reduce the stroke risk in those with poorer vitamin status.

There have been previous observational studies showing the positive strong association between HHcy and the risk of osteoporotic fractures,^(42–44)^ but the results from the intervention studies have been mostly negative.^(45–48)^ At present, although there is no clear explanation for this discrepancy, several possible underlying mechanisms have been suggested. For example, the dosage of B vitamins employed for the intervention greatly differs from one study to another, and mandatory food fortification of folate may have affected the results. Thus, the role of HHcy for the fracture risk awaits further investigation.

A recent study reported that vitamin B_6_, folate, and vitamin B_12_ intakes are partially correlated with improved functional outcome measurements in Dutch older adults.^(49)^ In our recent study for institutionalized elderly, we have reported that insufficiency of vitamin B_12_ and folate is a risk for decreased muscle strength.^(50)^ Most non-vertebral osteoporotic fractures occur at falling. Thus, HHcy increases the fracture risk both through decreased bone strength and increased risk of falling through muscle weakness.

With the ageing society, the number of patients with dementia is rapidly increasing causing societal burden, and identifying its modifiable risk factors is of great significance. The role of nutrition in the prevention of cognitive decline is receiving increasing attention,^(51)^ and the association of HHcy and cognitive decline and dementia has been reported.^(52,53)^ Recently published international consensus statement has concluded that HHcy is a modifiable risk factor for the cognitive decline and dementia in the elderly with the RR of 1.15 to 2.5 and the population attributable risk of 4.3 to 31%, and lowering plasma Hcy level with the intervention with B vitamins slows the cognitive decline.^(54)^ Clinically, the association of HHcy with vascular and non-vascular lesions have been described.^(55)^ Magnetic resonance imaging (MRI) has identified the cortical hippocampal atrophy, which is the regions affected in Alzheimer’s disease. The presence of white matter lesions and lacunar infarcts suggest the involvement of vascular lesions, since they are the pathologic findings in the small vessel lesions in the brain. Both vascular and non-vascular underlying mechanisms have been suggested for the detrimental effects of HHcy on the cognitive impairment. Possible non-vascular mechanisms include neurotoxic effects of HHcy such as the neuronal injury by the oxidative stress. Besides HHcy, the possible roles of other B vitamins in the pathogenesis of cognitive decline have been suggested including vitamin B_1_ and B_2_.^(56)^

## Vitamin B_1_

Vitamin B_1_ (thiamine) is one of the water-soluble vitamins, and serves as a cofactor in various metabolic pathways in the form of thiamine diphosphate (thiamine pyrophosphate; TPP). Vitamin B_1_ is involved in many oxidation-reduction reactions, such that it is a coenzyme for pyruvate dehydrogenase, alfa-ketoglutarate dehydrogenase, transketolase, branched chain α-keto acid dehydrogenase complex (BCKDH), and 2-hydroxyacyl CoA lyase.^(57,58)^ Thus, various aspects of metabolism are disturbed by vitamin B_1_ deficiency.

Vitamin B_1_ has no specific binding protein in the circulation, and it is easily filtrated in the glomerulus and excreted in the urine, and the bodily storage of vitamin B_1_ is quite limited with the maximum capacity of only 30 mg.^(57,58)^ Therefore, the depletion of its storage occurs within only two weeks of vitamin B_1_-deficient diet.

Vitamin B_1_ deficiency causes Wernicke’s encephalopathy (Wernicke-Korsakoff syndrome) and beriberi. The former is characterized by the abnormal eye movement, gait ataxia, and cognitive impairment, also amnesia and infusion in severe cases. The latter is subdivided into dry beriberi and wet beriberi. In dry beriberi, peripheral neuropathy such as impaired sensory and motor neuronal conduction occurs. Wet beriberi involves the cardiovascular system with such signs and symptoms as edema, tachycardia, cardiomegaly, and congestive heart failure.^(59,60)^ Defective above-mentioned enzyme activities engaged in energy metabolism result in insufficient ATP production. Since heart is the organs with high energy consumption, it is quite conceivable that cardiovascular involvement, especially heart failure, is the main manifestation of wet beriberi.

Previously, beriberi was quite prevalent in Japan. Since vitamin B_1_ is an essential cofactor in energy metabolism, it is quite natural that vitamin B_1_ deficiency causes impaired myocardial function and heart failure. With the discovery of vitamin B_1_ as well as the nutritional improvement of the society, the number of beriberi patients has dramatically decreased. Today, it seems to be generally considered that beriberi is a historical disorder and vitamin B_1_ deficiency is no longer a serious health problem in Japan.^(60–62)^

**Fig. 4:**
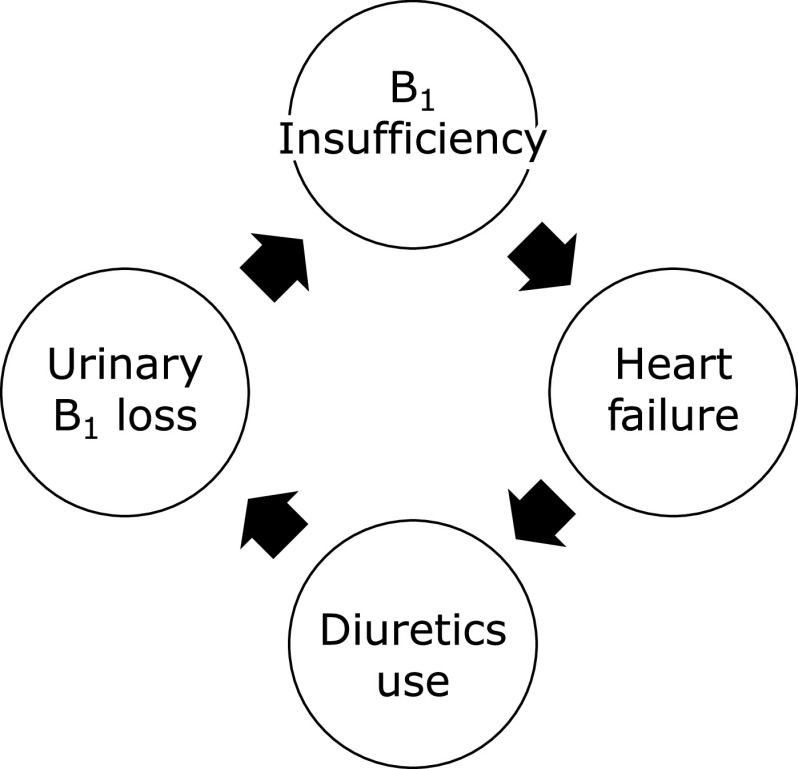
Hypothesis. Vicious cycle between vitamin B_1_ insufficiency and congestive heart failure.

Recently, the prevalence of vitamin B_1_ deficiency/insufficiency has been reported to be high in heart failure.^(63,64)^ There are several reasons rendering the patients with heart failure susceptible to vitamin B_1_ deficiency/insufficiency. Their vitamin B_1_ intake may be reduced due to splanchinic congestion and cachexia. Additionally, diuretics often used for the treatment of heart failure enhance the urinary loss of vitamin B_1_. However, reports on the association of vitamin B_1_ deficiency/insufficiency have been quite scarce in Japan, and we have considered it worthwhile to study the possibility that even vitamin B_1_ insufficiency can be a risk for heart failure. We have recently reported that plasma BNP (brain natriuretic peptide) concentration, which is a sensitive marker for heart failure, exhibited a significant inverse relationship with blood vitamin B_1_, which was confirmed by logistic regression analysis after adjusting for the various confounding variables in the institutionalized elderly. Blood vitamin B_1_ level was even lower in subjects with diuretics use (Fig. 4).^(65)^

The subjects in the current study were not severely vitamin B_1_ deficient, but blood vitamin B_1_ level was a significant risk factor for heart failure independent of age, BMI, and renal function. With the ageing society, the prevalence of elderly heart failure is rapidly increasing, and has become a serious societal problem.^(66)^ From our above data, it was considered likely that vitamin B_1_ insufficiency is a modifiable risk factor for elderly heart failure, and vitamin B_1_ can play an important role both clinically societally for its primary prevention.

## Societal Implication of Vitamin Insufficiency

When discussing the treatment effects for diseases, two endpoints are distinguished; true endpoint and surrogate endpoint. In the case of dyslipidemia, improvement of serum lipid levels and the prevention of cardiovascular diseases would be the example for the surrogate and true endpoint, respectively. For diabetes, the improvement of plasma glucose level and HbA1c, and the prevention of chronic complications would be the example of the former and the latter, respectively. Although true endpoint is superior to the surrogate endpoint, it requires much more time and research budget than the surrogate one.

Recently, breakthrough drugs with robust evidence for true endpoint have been developed for various diseases. For example, in the case of osteoporosis, bisphosphonates, which are potent inhibitor of bone resorption, improve the surrogate endpoint such as the bone mineral density, and markedly decrease the incidence of fragility fracture. Nutritional therapy including the one by vitamins is not so potent as the treatment effects of novel breakthrough drugs, which does not preclude the importance of nutritional therapy.

Intervention by drugs to subjects with high risk for fracture is cost-effective. However, because of the limited number of subjects belonging to the high-risk group, absolute number of fractures prevented is limited. In contrast, those in the low to intermediate risk comprise most of the population, and the absolute number of fractures is large, despite the lower risk in each subject. Drug intervention for low to intermediate-risk subjects is unsuitable considering the cost and the concern for the adverse events.^(67,68)^ A previous systematic review has reported that oral nutritional supplements use in the community produce an overall cost advantage or near neutral balance, often in association with clinically relevant outcomes, suggesting cost effectiveness.^(69)^ Then, prevention through lifestyle modification such as nutrition and exercise should be considered. Thus, vitamin insufficiency is a significant risk for various diseases, and its correction can be of great clinical and societal significance.

## Conclusion

It is a general belief that vitamin deficiency is mostly overcome in Japan, and the significance of vitamins in health promotion is receiving little concern. However, vitamin insufficiency, which is milder than deficiency, is a risk for various diseases (Table 1). Percentage of subjects with vitamin insufficiency is quite high, and much more dosage of vitamins is required for the prevention of insufficiency than for the prevention of deficiency. Vitamins are expected to play important roles in the prevention of various chronic diseases, and more clinical and epidemiological studies are needed.

## Author Contributions

MA and AK have collected and critically reviewed the papers on B vitamins, and vitamin D, respectively. KT has integrated the information and written the manuscript, which was revised several times after discussing with MA and AK.

## Figures and Tables

**Table 1:** Deficiency and Insufficiency of vitamins

Vitamin	Deficiency	Risk related to Insufficiency
Vitamin D	Rickets Osteomalacia	Fracture Falls (evident) Coronary heart diseases (possible) Cancer (possible) Upper respiratory tract infection (possible) Mortality (possible)
Vitamin B_12_	Megaloblastic anemia Damage to central nervous system	(through Hyperhomocysteinemia) Risk of cardiovascular diseases (especially stroke) Fractures Impaired physical function Cognitive decline
Folate	Megaloblastic anemia	
Vitamin B_6_	Seborrheic dermatitis Microcytic anemia Epileptiform convulsions Depression Confusion	
Vitamin B_1_	Wernicke-Korsakoff syndrome Beriberi	Heart failure
